# Surveillance of Broad-Spectrum Antibiotic Prescription in Singaporean Hospitals: A 5-Year Longitudinal Study

**DOI:** 10.1371/journal.pone.0028751

**Published:** 2011-12-09

**Authors:** Yi-Xin Liew, Prabha Krishnan, Chay-Leng Yeo, Thean-Yen Tan, Siok-Ying Lee, Wan-Peng Lim, Winnie Lee, Li-Yang Hsu, Network for Antimicrobial Resistance Surveillance (Singapore)

**Affiliations:** 1 Department of Pharmacy, Singapore General Hospital, Singapore, Singapore; 2 Department of Laboratory Medicine,Tan Tock Seng Hospital, Singapore, Singapore; 3 Department of Pharmacy, National University Health System, Singapore, Singapore; 4 Department of Laboratory Medicine, Changi General Hospital, Singapore, Singapore; 5 Department of Pharmacy, Khoo Teck Puat Hospital, Singapore, Singapore; 6 Department of Pharmacy, Tan Tock Seng Hospital, Singapore, Singapore; 7 Department of Medicine, National University Health System, Singapore, Singapore; The George Washington University Medical Center, United States of America

## Abstract

**Background:**

Inappropriate prescription of antibiotics may contribute towards higher levels antimicrobial resistance. A key intervention for improving appropriate antibiotic prescription is surveillance of prescription. This paper presents the results of a longitudinal surveillance of broad-spectrum antibiotic prescription in 5 public-sector hospitals in Singapore from 2006 to 2010.

**Methodology/Principal Findings:**

Quarterly antibiotic prescription data were obtained and converted to defined daily doses (DDDs) per 1,000 inpatient-days. The presence of significant trends in antibiotic prescription over time for both individual and combined hospitals was tested by regression analysis and corrected for autocorrelation between time-points. Excluding fluoroquinolones, there was a significant increase in prescription of all monitored antibiotics from an average of 233.12 defined daily doses (DDD)/1,000 inpatient-days in 2006 to 254.38 DDD/1,000 inpatient-days in 2010 (Coefficient = 1.13, 95%CI: 0.16–2.09, p = 0.025). Increasing utilization of carbapenems, piperacillin/tazobactam, and Gram-positive agents were seen in the majority of the hospitals, while cephalosporins were less prescribed over time. The combined expenditure for 5 hospitals increased from USD9.9 million in 2006 to USD16.7 million in 2010.

**Conclusions/Significance:**

The rate of prescription of broad-spectrum antibiotics in Singaporean hospitals is much higher compared to those of European hospitals. This may be due to high rates of antimicrobial resistance. The increase in expenditure on monitored antibiotics over the past 5 years outstripped the actual increase in DDD/1,000 inpatient-days of antibiotics prescribed. Longitudinal surveillance of antibiotic prescription on a hospital and countrywide level is important for detecting trends for formulating interventions or policies. Further research is needed to understand the causes for the various prescription trends and to act on these where necessary.

## Introduction

Antibiotics are an invaluable shared resource that is at risk of becoming both scarce and less effective in the face of a diminishing antibiotic pipeline and rising antimicrobial resistance [1]. A key component for improving appropriate antibiotic prescription is surveillance of prescription, and this may also indirectly contribute towards reducing antimicrobial resistance via establishment of national or institutional benchmarks, and heightening awareness [2], [3]. Information obtained from longitudinal surveys may also help identify trends in antibiotic prescribing and thus guide policy. Combined with similar surveillance of antimicrobial resistance, correlations between specific antibiotic prescription and resistance trends may be made that can help pinpoint intervention targets [4]–[6].

High antimicrobial resistance rates are prevalent in Singaporean hospitals [6], [7], resulting in widespread prescription of broad-spectrum antibiotics that in turn may select out even more antimicrobial-resistant pathogens [6]. Moreover, the proportion of inappropriately prescribed antibiotics is not low, with up to 40% of carbapenem prescriptions found to be inappropriate in a recent point-prevalence study involving three major local hospitals [8]. The Network for Antimicrobial Resistance Surveillance (Singapore) was established in 2006 to conduct laboratory- and pharmacy-based surveillance of antimicrobial resistance and antibiotic prescription in local public sector hospitals. The aim was to collate reliable data at institutional and national level, and analyze their trends over time, ultimately developing a tool that would allow for comparison with a national benchmark. This paper presents the results of the longitudinal surveillance of broad-spectrum antibiotic prescription from 2006 to 2010. A portion of the results from 2006 to 2008 had previously been analyzed in conjunction with Gram-negative resistance data, where relatively few correlations were found between prescription and resistance [6].

## Results

**Figure 1:**
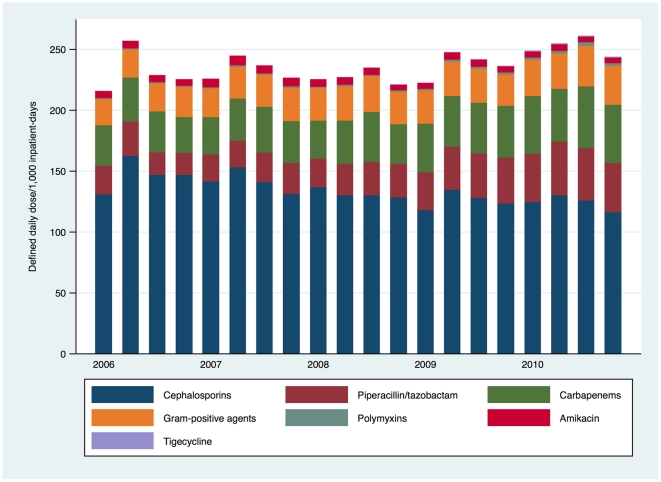
Prescription trends for major antibiotic classes and broad-spectrum antibiotics in Singaporean hospitals, 2006-2010.

Broad-spectrum antibiotic prescription results according to antibiotic class are depicted in Figure 1, with significant trends in prescription over time by class and individual antibiotic highlighted in Table 1. There was a significant increase in prescription of all surveyed antibiotics excluding fluoroquinolones from an average of 233.12 defined daily doses (DDD)/1,000 inpatient-days in 2006 to 254.38 DDD/1,000 inpatient-days in 2010 (Coefficient = 1.13, 95%CI: 0.16–2.09, *p* = 0.025), although the correlation coefficient (R^2^) was low at 0.28. Inclusion of the fluoroquinolones – which as a class comprised more than 60% of all antibiotics monitored – rendered the results non-significant. The increase in prescription of carbapenems, piperacillin/tazobactam, polymyxins and Gram-positive agents was balanced by a decrease in cephalosporin prescription and non-significant trend towards reduction of prescription of fluoroquinolones.

**Table 1:** Significant trends in antibiotic prescription in all Singaporean hospitals, 2006 to 2010.

Antibiotic	Trend	Coefficient	95% confidence interval	*p*-value	R^2^
All carbapenems	Increasing	0.931	0.608–1.255	<0.001	0.684
Ertapenem	Increasing	0.461	0.279–0.644	<0.001	0.625
Imipenem	Decreasing	−0.357	−0.572 – −0.141	0.003	0.418
Meropenem	Increasing	0.810	0.565–1.056	<0.001	0.741
All cephalosporins	Decreasing	−1.752	−2.204 – −1.300	<0.001	0.797
Ceftriaxone	Decreasing	−0.946	−1.412 – −0.480	<0.001	0.503
Ceftazidime	Decreasing	−0.370	−0.463 – −0.277	<0.001	0.806
Piperacillin/tazobactam	Increasing	1.43	0.919 – 1.946	<0.001	0.671
Gram-positive agents	Increasing	0.412	0.263 – 0.561	<0.001	0.666
Vancomycin	Increasing	0.191	0.043 – 0.340	0.015	0.302
Linezolid	Increasing	0.132	0.080 – 0.183	<0.001	0.631
Polymyxins	Increasing	0.099	0.062 – 0.135	<0.001	0.658

**Figure 2:**
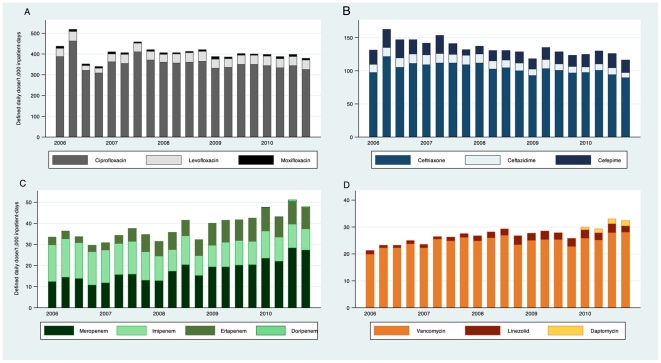
Prescription trends for individual antibiotics within major classes of broad-spectrum antibiotics in Singaporean hospitals, 2006–2010. (A) Fluoroquinolones. (B) Third and fourth generation cephalosporins. (C) Carbapenems. (D) Gram-positive agents.

The trends in prescription of individual antibiotics within major antibiotic classes are depicted in Figure 2. In line with what was previously described, imipenem prescription continued to decrease while meropenem and ertapenem prescription increased. Ciprofloxacin remained by far the most common antibiotic prescribed among those monitored, although prescription trends were stable from 2006 to 2010. Linezolid prescription increased significantly from 2006, but remained a fraction of vancomycin prescription. Newly introduced antibiotics such as tigecycline, daptomycin and doripenem were rarely prescribed.

Table 2 shows the trends in prescription among individual hospitals. In general, these mirrored the combined results, with increased prescription of carbapenems, piperacillin/tazobactam, and Gram-positive agents in at least 3 hospitals, while cephalosporins were less prescribed over time. Hospital 5, the smallest hospital, saw no significant change in prescription trends for any antibiotic.

**Table 2:** Trends in antibiotic prescription in individual Singaporean hospitals, 2006 to 2010.

Antibiotic	Hospital 1	Hospital 2	Hospital 3	Hospital 4	Hospital 5
All carbapenems	-	Increasing	Increasing	Increasing	-
Ertapenem	Increasing	Increasing	Increasing	-	-
Imipenem	Decreasing	-	-	-	-
Meropenem	Increasing	Increasing	-	Increasing	-
All cephalosporins	-	Decreasing	Decreasing	-	-
Ceftriaxone	-	-	Decreasing	-	-
Ceftazidime	Decreasing	Decreasing	Decreasing	-	-
Cefepime	-	-	-	-	N.A.*
Piperacillin/tazobactam	Increasing	Increasing	Increasing	-	-
Gram-positive agents	-	-	Increasing	Increasing	-
Vancomycin	-	-	Increasing	Increasing	-
Linezolid	-	-	-	Increasing	-
All fluoroquinolones	Decreasing	-	-	Increasing	-
Ciprofloxacin	Decreasing	-	-	Increasing	-
Levofloxacin	-	Increasing	-	N.A.*	-
Moxifloxacin	-	N.A.*	N.A.*	-	N.A.*
Amikacin	-	Decreasing	-	-	-
Aztreonam	-	-	-	Increasing	-
Polymyxins	Increasing	-	Increasing	Increasing	-

*N.A.  =  not available

**Figure 3:**
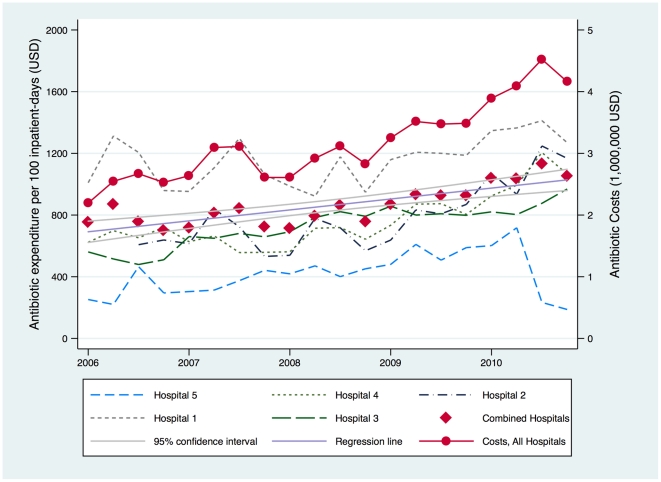
Trend in expenditure on broad-spectrum antibiotics in Singaporean hospitals, 2006–2010.

Trends in actual expenditure on antibiotics monitored per 100 inpatient-days are shown in Table 3 and Figure 3. The combined expenditure for 5 hospitals increased from USD9.9 million in 2006 to USD16.7 million in 2010, with a similar scale of increase seen in Hospitals 2-4.

**Table 3:** Trends in expenditure on surveyed antibiotics in Singaporean hospitals, 2006 to 2010.

Hospital	Trend	Coefficient	95% confidence interval	*p*-value	R^2^
Hospital 1	Stable	12.61	−3.52 – 28.73	0.117	0.138
Hospital 2	Increasing	35.14	12.51 – 57.77	0.005	0.422
Hospital 3	Increasing	22.78	14.57 – 30.98	<0.001	0.669
Hospital 4	Increasing	30.15	10.44 – 49.95	0.005	0.380
Hospital 5	Stable	4.22	−15.41 – 23.86	0.656	0.012
Combined Hospitals	Increasing	19.81	9.24 – 30.37	0.001	0.479

## Discussion

This work adds to the available sparse literature on country-level reports of hospital antibiotic consumption [9]–[11]. The surveillance program provides participating hospitals useful data for monitoring broad-spectrum antibiotic prescription and allows for benchmarking at the individual hospital level. The results of our surveillance call attention to the extensive prescription of broad-spectrum antibiotics in Singaporean hospitals. This is much higher compared to results from European hospitals [9]–[11], but is similar to those from a neighboring Asian hospital [12]. Part of this may be attributable to the high rates of antimicrobial resistance in the region [6], [7], [12], [13] that in turn drive up prescription of Gram-positive agents, carbapenems and polymyxins respectively.

However, local rates of methicillin-resistant *Staphylococcus aureus* (approximately 40% of all *S. aureus*), extended-spectrum beta-lactamase (ESBL)-producing Enterobacteriaceae (approximately 20% and 35% of all *Escherichia coli* and *Klebsiella pneumoniae* respectively) and carbapenem-resistant *Pseudomonas aeruginosa* (approximately 9% of all *P. aeruginosa*) have not increased over the five-year period. It is plausible that with greater awareness of these pathogens, clinicians were progressively prescribing more broad-spectrum antibiotics despite the lack of change in resistance trends. The issue of inappropriate prescription had been examined recently – the results with regards to carbapenem usage are consistent with findings in hospitals elsewhere [8].

Several antibiotics are natural substitutes of each other. Third- and 4^th^-generation cephalosporin prescription decreased even as piperacillin/tazobactam prescription increased in three hospitals. In Hospital 3, this had been actively encouraged in an effort to decrease the rates of ESBL-producing Enterobacteriaceae by decreasing cephalosporin selection pressure. The cause(s) for the switch in the other hospitals is less clear, although more aggressive marketing by the pharmaceutical industry and/or the direct impact of antimicrobial stewardship programs (ASPs) that had been established in Hospitals 1-3 between 2008 and 2009 cannot be ruled out as contributing causes. Ertapenem and meropenem prescription had increased at the cost of imipenem prescription – dosing advantages in the former and the perceived advantage of reduced neurological adverse effects in the latter had impelled the shift in prescription trends. In Hospitals 1 and 3, ertapenem had been promoted over the other carbapenems because of its lack of selective pressure on carbapenem-resistant non-fermenting Gram-negative bacteria [14].

The increase in expenditure on monitored antibiotics over the past 5 years reflects the actual increase in DDD/1,000 inpatient-days of antibiotics prescribed. The primary reason is that there was an increase in prescription of more expensive antibiotics (such as carbapenems, linezolid and piperacillin-tazobactam) whereas prescription of some of the cheaper generic antibiotics (ciprofloxacin, ceftriaxone and ceftazidime) had decreased. Among the three hospitals with ASPs, broad-spectrum antibiotic expenditure failed to increase only in Hospital 1, but this was the only institution where the ASP had covered all inpatient wards by 2010 [15]. Hospital 5 saw reduced patient numbers and antibiotic prescription in the first half of 2010 as it wound down its operations.

There are a number of limitations of this work. Firstly, we did not correlate antibiotic usage with resistance. However, this had been attempted previously with few significant results [6], and there is always a concern that multiple cross-correlations between antibiotic prescription and resistance will result in findings that arise purely by chance. Secondly, we only monitored prescription at a hospital-wide level and were therefore unable to drill down to target the units in the hospitals that were responsible for prescribing the bulk of these antibiotics. Thirdly, we had monitored antibiotic prescription trends purely in terms of DDD, and comparison with other forms of measurement such as prescribed daily doses have yielded discrepant results [16]. Nonetheless, for such macroscopic trending, DDD as unit of measurement is likely to be sufficient. Fourthly, narrow-spectrum antibiotics were not surveyed, and these results could have provided insight into the possible substitution of broad- for narrow-spectrum antibiotics. Beyond this, however, the additional utility of such data is unclear. Lastly, the nature of database surveillance is such that further work is required to determine and understand the causes for these trends, as by themselves, the paucity of data precludes further analysis.

In conclusion, longitudinal surveillance of antibiotic prescription on a hospital and countrywide level is important for detecting trends for the ultimate basis of formulating interventions or policies. Further research is needed to understand the causes for the various prescription trends and to act on these where necessary. It is important to note that both surveillance without intervention and intervention without surveillance are inefficient practices.

## Materials and Methods

This was a longitudinal multicenter database surveillance study carried out over a period of 5 years from 2006 to 2010. Five of 6 public sector hospitals in Singapore participated in the study – the sixth was a maternal and child hospital that was excluded because the large pediatric population would affect measurements of antibiotic prescription. There were 2 tertiary hospitals (Hospitals 1 and 2) and 3 secondary hospitals (Hospitals 3-5) ranging between 400 to 1,600 beds in size.

Antibiotic prescription data were extracted from each hospital's electronic pharmacy records. These figures comprise actual prescription data rather than purchase data. The antibiotics tracked included the carbapenems (imipenem, meropenem, doripenem and ertapenem), 3^rd^ and 4^th^ generation cephalosporins (ceftriaxone, ceftazidime and cefepime), fluoroquinolones (ciprofloxacin, levofloxacin and moxifloxacin), β-lactam/β-lactamase inhibitors (piperacillin/tazobactam), Gram-positive agents (vancomycin, linezolid and daptomycin) and others (amikacin, tigecycline and polymyxins). Denominator data in the form of hospital inpatient-days, i.e. the sum of each daily inpatient census every quarter, were obtained from each hospital's administrative records. The defined daily dose (DDD) per 1,000 inpatient-days for each drug and drug category prescribed every quarter was calculated following the World Health Organization (WHO) anatomical therapeutic chemical (ATC) classification of 2011 [17]. Drug expenditure was calculated based on cost price (in United States dollar at the current exchange rate) adjusted per 100 inpatient-days. The total broad-spectrum antibiotic expenditure for combined hospitals was also tabulated.

All statistical analyses were performed using Stata 11.0 (Stata Corporation, College Station, TX, USA). The presence of significant trends in antibiotic prescription over time for both individual and combined hospitals was tested by regression analysis and corrected for autocorrelation between time-points by using the Cochrane-Orcutt estimation following determination of the Durbin-Watson statistic. A trend was considered significant if the regression analysis showed a *p-*value of ≤ 0.05 and coefficient of determination (R^2^) greater than 0.3. Because tigecycline, daptomycin and doripenem were launched in Singapore within this period (2006, 2008, 2010 respectively) and prescription volumes were low, these antibiotics were not analyzed individually.
